# Gender role in anxiety, depression and quality of life in chronic kidney disease patients

**DOI:** 10.12669/pjms.36.2.869

**Published:** 2020

**Authors:** Um-e-Kalsoom

**Affiliations:** Um-e-Kalsoom, Ph.D. Assistant Professor, Department of Psychology, Shaheed Benazir Bhutto Women University, Peshawar, Pakistan

**Keywords:** Anxiety, Chronic kidney diseases, Depression, Gender, Perceived social support quality of life

## Abstract

**Objective:**

To investigate gender role in quality of life, anxiety, and depression in chronic kidney disease patients. The study hypothesized that men will score high on depression and, anxiety and will score low on quality of life and perceived social support as compare to women

**Methods:**

One hundred patients with chronic kidney diseases were selected using purposive sampling strategy from nephrology ward of Leady Reading Hospital, Peshawar. The participants were included both male (n=50) and female (n=50) ranging in age from 18-65 years (M=44.16, SD= 15.15) Patients were recruited on the basis of preset inclusion criteria i-e (willing to participat, could read, speak and understand urdu language, with no previous history of dialysis treatment) and exclusion criteria (Age > than 18, Previous psychiatric history, Diagnosis less than one year, Education less than high school). All participants completed Quality of Life Questionnaire, Anxiety and depression questionnaires.

**Results:**

Statistical analysis of independent sample t-test shows significant difference of men and women on QOL t(98)=2.34,p=.021), anxiety t(98)=4.23,p=.001), and depression t(98)=4.54, p=.001) while no significant gender differences were reported on perceived social support t(98)= .98,p= .327.

**Conclusion:**

Male showed more depression, anxiety as compared to females while women reported poor quality of life as compared to men.

## INTRODUCTION

Chronic kidney diseases (CKD) are influencing a huge number of individuals throughout the world. With expanding rate and predominance CKD influences 10– 15% of the adults worldwide.^1^ CKD causes gigantic misfortunes, yet in addition triggers significant difficulties concerning wellbeing. Wen and colleagues^2^ found that CKD patients had 83% higher rate of all-cause mortality and were twice as prone to kick the bucket from cardiovascular ailment. Because of the irreversible nature and less fortunate prognostic result, mental disorders are habitually normal among them. Patients with CKD are required to make continuous mental alteration through the span of their illnesses, for example, tolerating the life-threatening diagnosis and requirement for deep rooted treatment, learning dialysis systems, adapting to treatment disappointments, side effects and inconveniences.^3^ In the light of such a significant and continued ailments load, the administration of CKD/ESRD has extended from carefully clinical end point toward support of quality of life (QOL), from diagnosis to finish of life care.

Because of the above cited mental conditions of the patients with CKD, depression and anxiety are the essential mental issues of end-stage renal diseases (ESRD). Diagnosis of psychiatric disorders has been increasingly expanding as a definitive proportion of psychopathology in ESRD populace. Hence, it is vital to comprehend the collaboration of such mental comorbidities and CKD results.^4^ A study found frequencies of severity of depressive symptoms in patients who were having hemodialysis between 19% and 68%.^5^ Another study from Nigeria^6^ with the patients (N=118) with CKD proved development of depressive symptoms as 34.5% in patients who were on qualitative analysis as compared to the patients who were not receiving dialysis patients (13.3%). During a study conducted in urban center, Brazil, patients with CKD (N=170), Andrade and Sesso^7^ found a rather higher frequency of depressive symptoms among patients in qualitative analysis (41.6%), compared to patients underneath conservative treatment (37.3%). Andrade et al.^8^ in another study have found no important distinction within the prevalence of depression among CKD patients within the totally different stages of illness. CKD and its treatment represent a significant stress for affected people and need a large social adaption.

Junier et al.^9^ reported prevalence of depression in CKD as compared to normal population. Cukor et al.^10^ investigated depression and anxiety among patients having treatment of hemodialysis. Another study conducted on CKD patients on pre dialysis and hemodialysis stages in Pakistan, suggested occurrence of depression and anxiety was high at hemodialysis rather than pre dialysis stage.^11^ Intensity of CKD and its association with other psychiatric disorders may affect quality of life (QOL) and perceived social support (PSS). Both variables have been extensively studied in order to investigate not only the prognosis but also to see its impact on developing depression, anxiety and other psychiatric disorders. However, the existing literature is not filling the gap in knowledge regarding gender role and its impact on anxiety, depression, QOL and PSS. Therefore, the present study aimed to find out gender difference on anxiety, depression, QOL, and perceived social support.

## METHODS

For the present study one hundred (N=100) patients suffering from chronic kidney diseases were selected. Both male (n=50) and female (n=50) with age range rage 18-65 years (M=44.96, SD=15.153) equally participated in the study. Data were gathered from nephrology ward of local government hospital Peshawar using purposive sampling technique.

### Exclusion criteria

Age > than 18, Previous psychiatric history, Diagnosis less than one year, Education less than high school.

### Inclusion criteria

Willing to participate in the study, referred from nephrologists, could read and understand Urdu language, and with no history of previous dialysis treatment.

Basic information Sheet was devised to get information e.g age, sex, education, family structure, and marital status, and previous physical and psychiatric history, diagnosis of the participants. Multidimensional Scale of Perceived Social Support (MSPSS) a questionnaire which comprised of 12 items of 4-point rating scale was designed.^12^ Cronbach’s alpha reliability of the present study was good i-e 0.89. WHO quality Of Life brief,^13^ formed on 26 items, covered 4 areas of life e.g physical well-being, mental well-being, societal relations and environment, cronbach’s alpha scores for the present study are sufficient i-e 0.76 Pakistan anxiety and depression questionnaire (PADQ).^14^ To find out depression and anxiety symptoms an indigenous questionnaire was designed by Mumford et al. In the present scale 14 items were for depressive symptoms and 15 items were for anxiety symptoms. Alpha reliability of the present study is near to sufficient i-e. 0.63 and 0.71 respectively.

For the present study sample was collected from in and out door patients from nephrology ward of local government hospital Peshawar. Permission was sought from the Medical director of the hospital after the approval from ethical committee of the university. This data was collected using non-probability purposive sampling technique. After taking permission for data collection, researcher regularly visited nephrology department and briefed study objectives to the patients. Those who gave informed consent were included. They were assured that the information will be confidential and all data will be used only for research purpose. The willing participants were given a booklet of questionnaires comprised of above-mentioned questionnaires. The researcher helped participants how to fill out questions.

## RESULTS

A total of 100 CKD patients were recruited at the time of data collection, 50% male and 50% female equally participated. The age range categories comprised in 25.9% were from (18-30) age range, 11.9% from (31-40), 24.8% were in (41-50) while 36.7% were falling in (50-65) age group. Most of the subjects completed certificate level education (93.1%) married (87.1%) living in nuclear family system (87.1%) unemployed 57.5% (including house wife) and previous physical illness history (78.2%).

To compare gender differences t-test was computed. The results of Table-II indicate significant gender differences on QOL, depression and anxiety (p<0.01) however, no significant difference reported on PSS (p>0.05).

**Table-I:** Characteristics of the sample (N=100).

Variables	N (%)
***Age***	
18-30	26(25.9)
31-40	12(11.9)
41-50	25(24.8)
50-65	37(36.7)
***Gender***	
Male	50(50)
Female	50(50)
***Education***	
School	94(93.1)
College	4(4)
University	2(2)
***Marital Status***	
Single	12(11.9)
Married	88(87.1)
***Family Structure***	
Joint	25(24.5)
Nuclear	88(87.1)
***Employment Status***	
Yes	42(41.6)
No	30(29.8)
House wife*	28(27.7)
***Past Physical illness***	
Yes	79(78.2)
No	22(21.8
***Family CKD History***	
Yes	19(18.8)
No	81(80.2)

**Table-II:** Mean differences comparison of man and women on QOL, anxiety, depression and Perceived Social Support.

	Men	Women		

	M(SD)	M(SD)	t(df)	P
QOL	84.76(7.726)	80.80(9.114)	2.344(98)	0.021
Depression	22.92(1.926)	20.94(2.402)	4.547(98)	0.001
Anxiety	25.10(2.306)	22.82(3.035)	4.230 (98)	0.001
PSS	69.32(11.03)	66.96(12.85)	.985(98)	0.327

Table II results reveals significant difference of men and women on QOL, depression, and anxiety. However, results show no gender difference on PSS.

## DISCUSSION

The present investigation assessed depression, anxiety, quality of life, and perceived social support in patients with chronic kidney diseases. A plethora of studies in Pakistan have explored different variables relationship with CKD, however, limited studies looked at the gender role and its relationship with depression, anxiety, QOL and PSS. In the present study, Results of this study show significant gender difference on depression, anxiety and QOL, whereas PSS values were not significant. Moreover, mean differences indicated that women QOL was poor as compared to men. Previous findings, in this regard, show mix results e.g., Acaray and Pinar^15^ reported females QOL was higher than males. On the other hand, Tel^16^ and Suet-Ching^17^ suggested females QOL were poor as compared to males. However, the p value (p> 0.19) shows no significant gender difference on PSS, while mean differences indicate that men perceived more social support and better QOL as compared to women. The present study findings are supporting reasons due to cultural differences. Our society is male dominating, and conservative where social roles are strictly defined. As our values are to give more respect to man, therefore, despite chronic illness they still perceived better social support and QOL as compared to women.

Men show more depression and anxiety as compared to women. The findings are not in line with the traditional track where women show more depression and anxiety symptoms as compared to men.^18^ Similarly, another study associate tendency of developing anxiety symptom with female and depression with males.^19^ Endris, Fikreyesus and Amare^20^ highlighted women with poor social support, lower income as a risk factor of depression in hemodialysis patients. However, a local study reported no gender differences on depression among hemodialysis patients.^21^ The present data were taken from the general hospital of the local city (Peshawar), where usually middle and lower social class visit. As previously discussed, our society has strict define social roles e.g., man is responsible for holding all financial responsibilities on the other hand women have to be responsible for domestic chores. Usually sharing each other responsibility is against norms therefore never encouraged. Men showing more anxiety, depression could be due to cultural differences, when they feel financial pressure instead of performing their social roles they get dependent on other family members. Another reason could be that CKD treatment is very expensive and we don’t have health insurance policies, which may lead them towards depression, anxiety.

## CONCLUSION

Gender plays its role in developing the symptoms of anxiety as well as depression,. Gender as being male also influences on PSS and QOL. It also revealed that collectivist and male dominant cultural values along with disease could be responsible for developing symptoms of anxiety and depression. These parameters also influences in PSS and QOL of the patients with CKD.
